# An evaluation of oncofertility decision support resources among breast cancer patients and health care providers

**DOI:** 10.1186/s12913-019-3901-z

**Published:** 2019-02-06

**Authors:** Brittany Speller, Amanda Sissons, Corinne Daly, Marcia Facey, Erin Kennedy, Kelly Metcalfe, Nancy N. Baxter

**Affiliations:** 1 Department of Surgery, Li Ka Shing Knowledge Institute, St. Michael’s Hospital, Toronto, Canada; 2 Institute of Health Policy, Management, and Evaluation, Dalla Lana School of Public Health, University of Toronto, Toronto, Canada; 3 Leslie Dan Faculty of Pharmacy, University of Toronto, Toronto, Canada; 4 Department of Surgery, Mount Sinai Health System, Toronto, Canada; 5 Lawrence S. Bloomberg Faculty of Nursing, University of Toronto, Toronto, Canada; 6 Women’s College Research Institute, Women’s College Hospital, Toronto, Canada

**Keywords:** Decision aids, Patient education material, Fertility, Breast cancer, Decision-making, Information needs, Evaluation

## Abstract

**Background:**

Cancer patients of reproductive age are at risk of infertility as a result of their treatment. Oncofertility decision support resources can assist patients with fertility decision-making before treatment yet available oncofertility resources contain varying levels of detail and different fertility options. The key information/sections needed in oncofertility resources remain unclear. To explore the information needs for oncofertility decision-making before cancer treatment, we aimed to evaluate existing oncofertility decision support resources with breast cancer patients and providers.

**Methods:**

We conducted 30 to 90-min interviews that included a survey questionnaire and open-ended questions with patients and providers between March and June 2016. Interviews were transcribed verbatim. Analysis involved descriptive statistics for survey responses and thematic analysis of qualitative data.

**Results:**

A total of 16 participants completed interviews. Key information perceived by most participants as necessary for fertility decision-making included tailored post-treatment pregnancy rates, cost ranges and financial assistance for the fertility options based on patients’ situation. However, patient and provider participants expressed differing opinions on the inclusion of all before and after treatment fertility options and the amount of fertility information required at diagnosis.

**Conclusion:**

The evaluation identified fertility information needs among patients in addition to providers’ views on patient needs. While existing oncofertility resources contain information perceived as necessary for decision-making there is an opportunity to use these findings to create or enhance resources to better meet the needs of patients. Additionally, patients and providers differing views on information needs highlight the opportunity for provider training to ensure better communication using resources in clinic to understand specific patient needs.

**Electronic supplementary material:**

The online version of this article (10.1186/s12913-019-3901-z) contains supplementary material, which is available to authorized users.

## Background

Cancer patients of reproductive age may experience compromised reproductive function in survivorship as a result of their treatment [1–4]. Accordingly, these individuals often need to make time-sensitive decisions about pursuing fertility interventions prior to commencing cancer treatments [3]. Fertility guidelines updated in 2018 by the American Society of Clinical Oncology (ASCO) recommend that infertility risks and fertility preservation (FP) options be discussed early with patients and referrals made to specialists and organizations with resources to facilitate fertility decision-making [5].

Decision support resources, such as decision aids, have been recommended as a supplement to discussions to assist patients with fertility decision-making [6–12]. However, the varying informational needs among patient populations based on their different knowledge levels and experience can create challenges for resource developers [13].

Oncofertility decision support resources for cancer patients of reproductive age are available in various countries and include interactive online tools, brochures, paper and online decision aids, and option grids [11, 14–18]. While oncofertility resources are available, they contain varying levels of detail, cover differing FP and parenthood options, and most do not directly include the option to forgo any fertility intervention. The key information needed for informed fertility-decision making, the content that is most valuable, and optimal formatting (e.g., online, paper, video), and dissemination of oncofertility resources to support patients and health care providers during fertility discussions and decision-making remains unclear. To explore the information needs for oncofertility decision-making before cancer treatment, we aimed to evaluate existing oncofertility decision support resources with breast cancer patients and providers.

## Methods

The Research Ethics Board (REB) at St. Michael’s Hospital in Toronto, Ontario approved the evaluation (REB#15–220) and ethics was obtained from recruiting sites. All participants provided written and verbal consent prior to participation in the evaluation.

### Selection of Oncofertility decision support resources

We reviewed the indexed and grey literature in consultation with oncology and fertility content experts, which identified six patient-focused oncofertility resources for evaluation by Canadian breast cancer patients and providers [15–19]. The six oncofertility resources included two decision aids from Australia [15] and the Netherlands [16], an option grid from Canada [18] and three patient educational materials from the United States [17, 19] (LIVESTRONG fertility booklet, LIVESTRONG Family-Building Option Tool, and the MyOncofertility educational website that is now SaveMyFertility) (Table 1). While other educational materials related to fertility are available for cancer patients [11, 14], the selected oncofertility resources represent a range of detailed resources (e.g., Australian and Dutch decision aids, MyOncofertility educational website), shorter resources (e.g., Canadian option grid, LIVESTRONG booklet and Family-Building Option Tool), and simple and complex resources (e.g. the option grid is a one page tool, the Australian decision aid is a booklet, and the Netherlands decision aid is an interactive online tool). The oncofertility resources contained similar sections (e.g. listing established FP options) but also had unique characteristics (e.g. an explicit values clarification method in the decision aids) (Table 2).

**Table 1:** Characteristics of the evaluated oncofertility decision support resources

Decision Support Resources		Decision Aids			Patient Educational Materials		
		Australian Decision Aid	Netherlands Decision Aid	Sunnybrook Option Grid	LIVESTRONG Booklet	LIVESTRONG FB^†^ Option Tool	MyOncofertility (now SaveMyFertility)
Resource Description	Author	Peate et al.	Garvelink et al.	Warner et al.	LIVESTRONG	LIVESTRONG	Oncofertility Consortium®
	Development Group	Academic Teaching Institution	Academic Teaching Institution	Academic Hospital	Non- Profit Organization	Non- Profit Organization	Private Research University
	Year Created/ Updated	2011/ 2016	2013/2014	2015	2013	–	2011
	Type	DA^†^ Booklet	DA^†^ Website	Online PDF Grid	Booklet	Online Tool	Educational Website
	Language	English	Dutch	English	English/Spanish	English	English/ Spanish
Target Population	Sex	Females	Females	Females	All	All	All
	Cancer Type(s)	Breast Cancer	Breast Cancer	Breast Cancer	All Cancer Types	All Cancer Types	All Cancer Types
	Country	Australia	Netherlands	Canada	United States	United States	United States

^†^*Abbreviations: DA* decision aid, *FB* family-building

**Table 2:** Sections and information included in the evaluated oncofertility decision support resources

Decision Support Resources Sections and Information		Decision Aids			Patient Educational Materials		
		Australian Decision Aid	Netherlands Decision Aid	Sunnybrook Option Grid	LIVESTRONG Booklet	LIVESTRONG FB^†^ Option Tool	MyOncofertility (now SaveMyFertility)
Background Information	Explanation of female fertility	✓	✓	–	–	–	✓
	Explanation of female infertility	✓	✓	–	✓	–	✓
	General factors that affect fertility	✓	✓	–	✓	–	✓
	Role of health care provider	✓	–	–	–	–	✓
Fertility Options Before Treatment	Wait and see	✓	✓	–	–	–	–
	Egg freezing	✓	✓	✓	✓	✓	✓
	Embryo freezing	✓	✓	✓	✓	✓	✓
	Ovarian tissue freezing	✓	✓	✓	✓	✓	✓
	Ovarian suppression	✓	–	✓	✓	✓	✓
	Ovarian transposition	–	–	✓	–	–	–
	Ovarian shielding	–	–	–	✓	✓	✓
	Fertility-sparing surgery (e.g., Trachelectomy)	–	–	–	✓	✓	–
	In vitro maturation	–	–	–	✓	✓	–
Parenthood Options After Treatment	No more children	✓	✓	–	–	–	–
	Egg donation	✓	✓	–	✓	✓	✓
	Embryo donation	✓	–	–	✓	✓	✓
	Surrogacy	✓	–	–	✓	✓	✓
	Adoption	✓	✓	–	✓	✓	✓
	Foster parenting	–	✓	–	–	–	–
	Natural conception/ Fertility testing	–	–	–	–	✓	–
Information on Cancer/ Support	Cancer diagnosis	✓	–	–	✓	–	✓
	Cancer therapies	✓	✓	–	✓	✓	✓
	Possible fertility outcomes after cancer or cancer therapies	✓	–	–	✓	–	✓
	Cancer/ therapies and impact on fertility	✓	✓	–	✓	–	–
	Cancer/ therapies and pregnancy or lactation	✓	✓	–	–	–	–
	Effects on family, children, and relationships	✓	✓	–	✓	–	✓
	Psychosocial concerns	✓	–	–	✓	–	✓
Other Sections	Values clarification method	✓	✓	–	–	–	–
	Personal stories	✓	–	–	✓	–	✓
	Health care provider directed questions	✓	✓	–	✓	–	✓
	Sources for more information	✓	✓	✓	✓	–	✓
	Notes section	✓	–	–	–	–	–
	References	–	✓	–	–	–	–
	Glossary	✓	–	–	–	–	–
	Length of the resource	66 pages (2011) 37 pages (2016)	5 chapters, 26 information pages	1 page	11 pages (6 pages female specific)	2 website pages, dropdown option grid for each option presented	5 chapters, 47 questions for females (patient section)

^†^*Abbreviations: FB* family-building

### Sample participants

We recruited breast cancer patients between the age of 18 and 45 who experienced fertility decision-making prior to their fertility-risking treatment within the past five years. Participants were recruited in person at two breast cancer clinics in the Greater Toronto Area and online through Canadian-wide advocacy groups and cancer organizations. Through the research team’s circle of contact and snowball sampling [20] multi-disciplinary health care providers who provide care to young breast cancer patients were also recruited from across Canada.

### Data collection

Interviews occurred between March 2016 and June 2016. Participants had the option to complete the interviews in person (for those located in the Greater Toronto Area, Ontario) or by telephone. A structured questionnaire, tailored to the unique features of the six oncofertility resources and role of participant (e.g., patient or provider) was used to conduct the interviews. The guide contained both closed-ended and open-ended questions, which allowed participants to reflect on their experiences and expand on their responses. Closed-ended questions were rated using multiple choice and different 5-point Likert scales depending on the set of questions. For example, participants rated the usefulness of each oncofertility resource section using the scale: 1 = not at all useful, 2 = not very useful, 3 = useful, 4 = very useful, 5 = not sure; participants then rated their level of agreement on the oncofertility resources usability and flow of content using the scale: 1 = strongly disagree, 2 = disagree, 3 = neither agree nor disagree, 4 = agree and 5 = strongly agree; finally participants rated the importance of factors that can impact fertility decision-making on a scale of: 1 = not at all important, 2 = not very important, 3 = important, 4 = very important, 5 = not sure. Participants were also asked to rate the length of oncofertility resources using the scale: 1 = too short, would prefer it to be much longer, 2 = short, would prefer it to be a bit longer, 3 = just right, 4 = long, would prefer it to be a little shorter, 5 = too long, would prefer it to be much shorter; as well as the use of figures in the oncofertility resources using the scale: 1 = too few, would prefer a lot more, 2 = few, would prefer a few more, 3 = just right, 4 = a lot, would prefer a few less, 5 = too many, would prefer a lot less. Questions focused on the content, usability, and design features of the oncofertility resources and the general use of resources (Additional file 1). Prior to the interviews, participants received two of the six oncofertility resources through random assignment (one decision aid and one patient educational material) by email to allow for familiarization of the resources prior to the interview. Evaluations continued until each oncofertility resource was reviewed by at least three participants.

### Data analysis

Interviews were audio recorded, transcribed verbatim, and audited by the interviewer to ensure content accurately reflected what was said in the interview. Responses to closed-ended questions were inputted into Microsoft Excel 2016 and frequencies were calculated for each question. Thematic analysis was used to analyze the open-ended responses [21]. Any elaboration by participants on the questions was deductively coded by two team members independent of each other using a coding scheme that reflected the sections and unique content of the oncofertility resources. NVivo 11.2.2 was used to organize the data and facilitate the qualitative analysis. Themes were developed based on common ideas across the six evaluated oncofertility resources and data repetition [21]. Team members and coders met regularly to discuss the coding, analysis, and emerging findings.

## Results

A total of 16 interviews were conducted, two in-person and 14 by telephone, with patient participants (*n* = 8) and provider participants (*n* = 8) (Table 3). Interviews ranged from 30 to 90 min with most participants evaluating two of the oncofertility resources; five participants only evaluated one resource due to time limitations. However, each oncofertility resource was evaluated by at least three participants.

**Table 3:** Patient and provider participant characteristics

Patient Participants Characteristics (*n* = 8)	N	Mean (SD)
Previous Children		
Yes	3	
No	5	
Race		
White	4	
Non-white	4	
Relationship Status at Diagnosis		
Married/Common-Law Marriage	5	
Long-Term Relationship	2	
Single	1	
Education		
Post-Secondary Schooling	4	
Completed or Enrolled in Graduate Level Studies	4	
Location		
Ontario	7	
Québec	1	
Age at Diagnosis	21 to 35 years	31 (4.6)
Time Since Diagnosis	1 to 4 years	2.25 (1.6)
Provider Participants Characteristics (*n* = 8)	N	Mean (SD)
Profession		
Fertility Specialists	2	
Oncology Health Care Providers (including a surgeon, oncologist, social worker, and nurse)	4	
Location		
Ontario	5	
British Columbia	2	
Manitoba	1	
Hospital Setting		
Community	2	
Academic	6	
Time working in field	4 to 30 years	15 (10.14)

### Utility of oncofertility decision support resource content and format

In general, patient and provider participants perceived the following sections included in the oncofertility resources to be useful (i.e. over 90% of participants rated these sections as ‘useful’ or ‘very useful’): the fertility options before treatment and parenthood options after treatment; availability of financial assistance and cost of each fertility option; the list of additional resources for more information and support; the question list for health care providers; and the glossary of terms. However, participants expressed varied responses on the perceived usefulness of some resource sections including: the option grids to summarize information; the background information sections; the personal stories; the values clarification methods in the decision aids; the ongoing research on fertility and cancer; and the videos, animations, and graphics (Table 4).

**Table 4:**
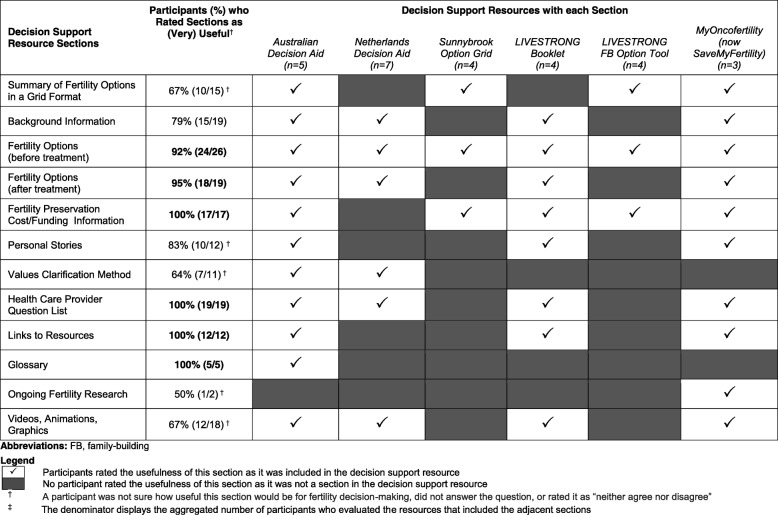
Sections participants rated as ‘useful’ or ‘very useful’ in the evaluated oncofertility decision support resources

Patient and provider participants also evaluated the usability, readability, and content of each oncofertility resource (Table 5). Most patient and provider participants rated the Australian decision aid (4/5 participants, 80%) and Netherlands decision aid (6/7 participants, 86%) as long or too long, and the LIVESTRONG Family-Building Option Tool as short or too short (4/4 participants, 100%). Patient and provider participants also thought that all oncofertility resources flowed in a logical order (6/6 resources, 100%) and indicated a preference for paper and/or online resources (14/16 participants, 88%) over other format options including audio-guided booklets or videos.

**Table 5:** Patient and provider participant agreement on the usability, readability and content in the evaluated oncofertility decision support resources

Decision Support Resources	Length of the resource (Just right)	Information easy to read (Strongly) Agree^b^	Information flows in a logical order (Strongly) Agree	Presentation of the fertility options is balanced (Strongly) Agree	Enough information to decide on a fertility option (Strongly) Agree	Resource is easy to use (Strongly) Agree	Training is needed for patients before using this resource (Strongly) Agree	Training is needed for health care providers before using this resource (Strongly) Agree	Resource would have been useful if it used (Strongly) Agree
Australian Decision Aid (*n* = 5)	1/5	4/5	5/5	4/5	3/5	4/5	2/4^a^	3/4^a^	4/5
Netherlands Decision Aid (*n* = 7)	1/7	5/7	6/7	5/7^a^	4/7^a^	5/7	2/7	3/7	6/7
Sunnybrook Option Grid (*n* = 4)	3/4	4/4	4/4	4/4	2/4	4/4	1/4	0/3^a^	4/4
LIVESTRONG Booklet (*n* = 4)	3/4	4/4	4/4	3/4	1/4	4/4	0/4	2/4	4/4
LIVESTRONG FB† Option Tool (*n* = 4)	0/4	4/4	3/4^a^	2/4	0/4^a^	4/4	1/4	2/4	2/4
MyOncofertility *(now SaveMyFertility)* (*n* = 3)	2/3	2/3	3/3	3/3	2/3	3/3	0/3	0/3	2/3

^†^Abbreviations: *FB* family-building

^a^One participant was not sure how useful this section of the decision support resource would be for fertility decision-making, did not answer the question, or rated it as “neither agree nor disagree” in the Likert scale

^b^(Strongly) Agree includes participants who answered ‘agree’ and ‘strongly agree’

Participants expressed varying opinions on whether the oncofertility resources contained enough information to make an informed fertility decision. Among the participants who reviewed the more detailed resources (e.g., the Australian and the Netherlands decision aids and MyOncofertility educational website) 60% (9/15 participants - eight patient participants and one provider participant) reported that there was enough information for decision-making, whereas only 18% (2/11 patient participants and no provider participants) reported that there was enough information for decision-making in the shorter resources (e.g., the option grid, LIVESTRONG fertility booklet, and LIVESTRONG Family-Building Option Tool). More patient participants (8/9, 89%) also perceived that the detailed resources contained enough information for decision-making, in comparison to provider participants (1/5, 20%).

### Preferences for fertility information

Four themes were discerned from the data: challenges on the delivery and use of fertility information in clinical practice; ideal delivery and timing of decision support resources in clinical practice; perspectives of information needs for informed fertility decision-making; and factors influencing FP decisions. Illustrative quotes from patient and provider participants for each theme are presented in Table 6.

**Table 6:** Themes discerned from the evaluation of oncofertility decision support resources and illustrative quotes from patient and provider participants

Themes	Description	Patient Participants	Provider Participants
Challenges on the delivery and use of fertility information in clinical practice	Fertility information	*“I feel that* [providers] *don’t want to give you as much information as maybe you would like, I know that they had suggested to me that they thought it would be overwhelming to give too much information and I feel for my personality it was the opposite. I didn’t have enough information. I might not have made the same decision actually…”* (Patient, 02)	*“…I do find it a little bit misleading some of the information that can be provided on resources…potentially having a baby in the future* versus *being alive is often a dilemma that our patients face…I don’t think that you can convey that kind of information very well in an online tool…there’s something about conveying that information in kind of a verbal way that I think is needed.”* (Provider, 06)
	Self-advocacy for fertility discussions	*“So basically it’s some information I found on the internet that I learned about the chemotherapy and the fertility issues so I had to bring it up myself to the oncologist.”* (Patient, 03)	
Ideal delivery and timing of decision support resources in clinical practice	Timing of resource delivery	*“…by the time* [fertility preservation] *was* [presented] *than everything was just crunched and everything seemed like a rush because…it was presented like when they had a treatment plan right. So that’s why I think it’s really important to get* [a resource] *like basically at diagnosis right or you know when they sit you down so that you can start thinking about it. And you have the time frames in front of you, so you know how it will affect your treatment plan.”* (Patient, 04)	*“… I think it would be great, if they would get* [a resource] *on day 1 essentially and really read it before they come to their fertility consult.”* (Provider, 01) *“I would say* [the idea time for a resource is] *when discussing their treatment plan, I think at diagnosis is too early. I often see women who have gone to fertility specialists…and they didn’t need it in the first place because they were never going to get chemo...”* (Provider, 06)
Perspectives of information needs for informed fertility decision-making	Background information	*“I found* [the resource] *really useful, I like the part where they said not all treatments could affect your ability to have kids and also if your period returns it doesn’t necessarily mean that your ovaries are as effective, because I think that sometimes is a misconception. So, I think it’s pretty good.”* (Patient, 06)	*“My feeling is that patients should have as little information as they absolutely need, they are completely overwhelmed with information so, I don’t actually think they need to know how chemotherapy destroys the ovaries, I think they believe us if we say it does, so I think it’s a bit more information than they need…”* (Provider, 03)
	After treatment parenthood options	*“…Even though* [adoption] *like, it’s really you don’t want to look at that option…but I think it’s good that it’s included because it just gives you like even if it’s something that you choose not to at least you kind of, you are aware of it…”* (Patient, 04)	*“… I think people hopefully know that they can adopt or foster children, or just not have children so, it’s not necessarily bad to have it in there but I think it’s maybe less useful.”* (Provider, 07)
	Value of option grids	“*I think the grid at first is great as a starting point and then if you do want more information something like a larger grid maybe or a website or a pamphlet regarding any additional options that are available with more detailed information.”* (Patient, 08)	*“… I definitely think* [the Option Grids are] *a good starting point though but, you know, there’s things that you are never going to build into a grid like…your partner or your support system, support, any kind of like emotional aspects that you are not really going to capture I don’t think, not that, nor should you but this is just sort of one piece of the puzzle.”* (Provider, 07)
Factors influencing FP decisions	Emotional support	*“I think the emotional support… I don’t think they realize how emotionally taxing it is and also how taxing it is on your body and then you say you’re going into treatment…we definitely need some more emotional support*.” (Patient, 02)	*“… with medical counselling* [patients] *will be presented* [their] *options based on* [their] *personal situation but then the choice from that point quite often is emotional.”* (Provider, 02)

#### Challenges on the delivery and use of fertility information in clinical practice

Patient participants noted that they did not receive enough fertility information when diagnosed with cancer. In instances where information was provided one patient participant described it as ‘piece meal’ and ‘dumped on my lap’. Another patient participant reported that more fertility information before treatment might have changed their final fertility decision. There was belief among patient and provider participants that too much information may overwhelm patients but some patient participants also noted that if fertility was important to them they would take the time to read the information provided. Following clinical appointments all patient participants indicated they had searched for more fertility information online and one used the information found online to initiate a fertility discussion with her oncology health care provider.

Among the provider participants, only a few (2/8, 25%) said they provided patients with oncofertility resources including ones developed specifically for their clinic and on FP financial assistance. The remaining provider participants said they provided verbal fertility information to their patients along with referrals to reproductive specialists. One provider commented that they did not provide oncofertility resources as the decision is often quite emotional for patients and therefore felt resources were unable to help with those emotions in the same way as medical counseling. In addition, a provider participant noted that some oncofertility resources contained misleading information on the fertility options. Role uncertainty was also discussed; some provider participants felt that it was not their role to provide oncofertility resources to patients and were unclear on who was most responsible to provide these resources.

#### Ideal delivery and timing of decision support resources in clinical practice

Patient and provider participants had mixed opinions on the appropriate time to deliver an oncofertility resource to patients. Some participants recommended that oncofertility resources be delivered as soon as the patient is diagnosed (3 patient participants and 3 provider participants, 38%). Others felt the ideal time point is when discussing the treatment plan (5 patient participants and 4 provider participants, 56%). A few provider participants expressed concerns about early presentation and referrals to reproductive specialists as not all cancer treatment is fertility-risking and patients are often initially overwhelmed by their cancer diagnosis and treatment information. However, it was noted that bringing up fertility information too late may also overwhelm patients. Patient and provider participants also expressed varying views on who should deliver an oncofertility resource; some indicated that the provider with whom the patient had the most rapport would be best; however it was also important that the resource was delivered by a provider who would not bias patients’ decision. In contrast, other participants felt an oncofertility resource should be completed with providers such as medical oncologists (2 patient participants and 2 provider participants, 25%) so that patients can receive specific information on their risks for infertility and/or nurses or social workers (3 patient participants and 2 provider participants, 31%) so patients can have access to psychological counseling as they make their decision.

#### Perspectives of information needs for informed fertility decision-making

**Fig. 1:**
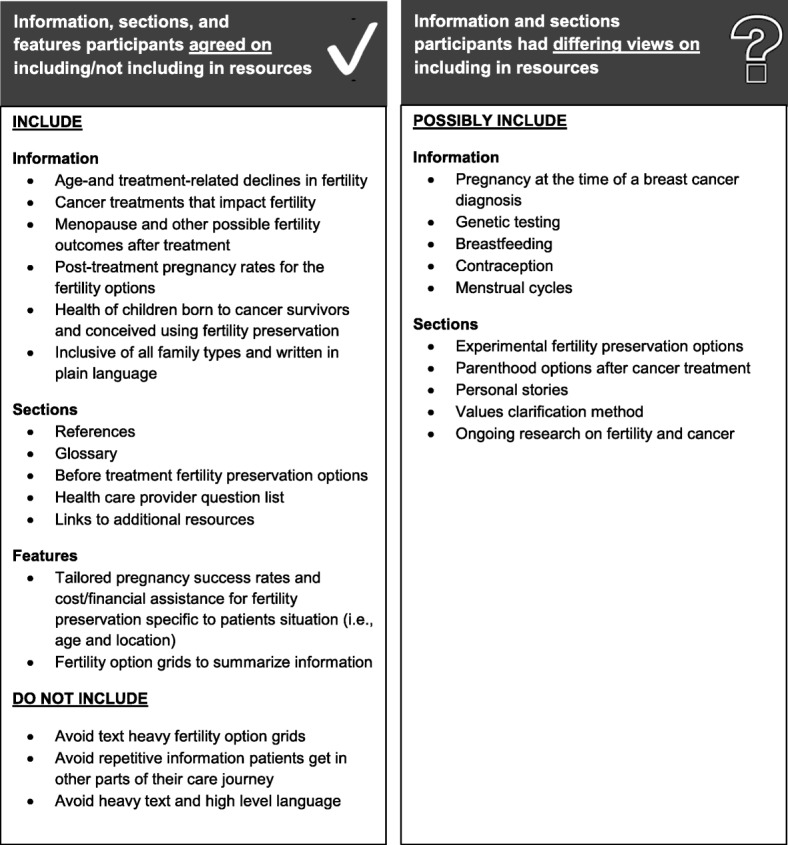
Summary of recommended information, sections, and features for inclusion in oncofertility decision support resources

Patient and provider participants identified the specific background and fertility-related information, sections, and features necessary for informed decision-making (Fig. 1). Information included: (1) age-and treatment-related declines in fertility; (2) cancer treatments that impact fertility; (3) menopause and other possible fertility outcomes after treatment; (4) post-treatment pregnancy rates with each fertility option; and (5) health of children born to cancer survivors and conceived using FP. The inclusion of accurate cost ranges for the FP options as well as financial assistance programs were noted as important to include to ensure patients are not shocked or disappointed following FP.

Patient and provider participants had differing opinions on the inclusion of other information in oncofertility resources including: (1) pregnancy at the time of a breast cancer diagnosis; (2) genetic testing; (3) breastfeeding; (4) contraception; and (5) menstrual cycles. Patient participants who did not have this information during their decision-making felt it was important to include in an oncofertility resource. On the other hand, provider participants noted that including information that did not directly contribute to fertility decision-making prior to treatment could cause more confusion or unrealistic expectations among patients. Mixed opinions were also noted on the inclusion of experimental FP options; patient participants preferred having all options listed while provider participants expressed concern that these options were not available universally across Canada. If experimental FP options were included participants stated that they should be presented after established FP options. Some participants suggested including fewer FP options upfront with other options as supplementary information for those who desired additional information. While most patient and provider participants felt parenthood options after cancer treatment would be useful to include, some provider participants thought it was too much information and felt most patients were knowledgeable on the options following treatment.

With respect to values clarification methods and personal stories in oncofertility resources, notably while many participants (64 and 83% respectively) rated them as useful, some expressed concerns with this information. Specifically, there was concern that personal stories might influence patients to choose the same option as the storyteller if similarities were present in their experiences. Provider participants also noted that an explicit values clarification method with many prelisted factors may overwhelm patients; however, patients preferred the prelisted factors to use as a starting point for their decision-making. While some patient participants found the values clarification method a useful way to break down and solidify their fertility decision, others felt that they were not in line with how they typically processed information and made decisions and therefore did not believe it would be useful. Additionally, while option grids with simple information were noted as a useful starting point for decision-making, provider participants advised that they do not encompass all the aspects of fertility decisions (e.g., emotional aspects of care) and thus were not considered adequate as a stand-alone resource for patients.

Overall, content in the oncofertility resources that repeated information already provided to patients during other parts of their care journey (e.g. prevalence and general breast cancer facts), or that used heavy text and high-level language was not seen to be of value by patient or provider participants. Patient and provider participants noted that content and graphics included in oncofertility resources should be diverse and inclusive of all family-types. Patient participants also wanted information on the pregnancy success rates and cost of FP tailored to their situation vs. general information. Most oncofertility resources evaluated lacked references (5/6, 83%), and this caused provider participants to have limited trust in the information, particularly when it differed from their understanding of the topic.

#### Factors influencing FP decisions

Many factors were seen to influence fertility decision-making by patients and providers. Key factors highlighted by participants included:

- Stage and severity of the cancer diagnosis
- Time required to complete FP
- Chance of a cancer recurrence
- Cost of the FP options
- Outcomes for children conceived using FP and pregnancy success rates of the FP options
- Desire for biological children
- Pregnancy at diagnosis and current children
- Patient’s age
- Relationship status
- Experience completing FP prior to the diagnosis
- Factors involved in the FP procedure (e.g., invasive component)
- Support (e.g., emotional) from providers and support person(s) (e.g., partners who may share in the decision-making on FP with patients)
- Distance from hospital to FP clinics
- General feelings of being overwhelmed

Common factors agreed on by most participants included the stage and severity of the cancer diagnosis, time required to complete FP, and the accurate cost of FP as the decision was perceived as more complex if no funding is available.

## Discussion

This study aimed to evaluate six oncofertility resources to explore, understand, and describe the fertility information needs among patients from the perspective of breast cancer patients with the experience of FP decision-making and providers. The use of existing oncofertility resources as a reference for participants allowed for a more thorough understanding of information needs as patient participants drew from past experiences and discussed information in the resources never mentioned to them but that they found valuable. To our knowledge, this is the first evaluation of patient-focused oncofertility resources with patient and provider participants. This evaluation provides insights into the specific information, sections, and formatting that should be considered when developing oncofertility resources for cancer patients of reproductive age. It also highlights that in general existing oncofertility resources contain information that meets the needs of young breast cancer patients; however, not all information in the resources was perceived as necessary for informed decision-making.

The fertility information needs among cancer patients in Australia has been explored prospectively by Peate et al. [22], and this evaluation found that in addition to general information on the impact of cancer treatment and fertility patients perceived factors impacting fertility such as age related declines are valuable when making fertility decisions. The findings from a qualitative study with young breast cancer patients by Thewes et al. [8], showed that patients had questions on the pregnancy rates after treatment, available fertility options, success rates of FP, risk of cancer recurrence after pregnancy and use of contraception, similar to participants in this evaluation. This evaluation also highlighted the perceived value of tailored pregnancy success rates and cost/funding information to the patients situation (e.g., age and location), and various components of oncofertility resources that are useful such as an option grid summary, additional resources, a glossary, a reference page to encourage trust in the information presented, and a health care provider directed question list.

Participants felt that supplementary information (e.g., experimental FP options) should be accessible based on each patient’s information needs due to the variability in needs between individuals. Our findings also show that information should be inclusive of various family types and avoid high-level language, which can be achieved by adhering to plain language best practices [23–25] to ensure understanding by patients. The factors influencing FP decisions found in this evaluation can be utilized with specific internal and external factors identified by other studies, such as Jones et al. [6], to create sections in oncofertility resources such as an explicit values clarification method.

Patient and provider participants expressed different opinions in the evaluation on the amount of information to include in oncofertility resources (for example on genetic testing and breastfeeding) and the inclusion of all before treatment and after treatment fertility options. Most patients generally prefer to receive information from their providers during their clinical encounters [26], and Peate et al.*,* note that many patients prefer to make fertility decisions after consideration of their providers opinions or through shared decision-making [22]. Therefore, differences in the perception of information needs by providers and patients may affect the quality of information presented to patients, limiting some patients’ ability to make a fully informed decision. The differing opinions highlight the potential role for enhanced communication in clinical settings with the use of oncofertility resources to ensure providers understand each patient’s information needs. Additionally, these different opinions can prove difficult when designing resources in general as developers often have to decide on whose views to utilize for the different sections [13, 27]. As such, there may be a role in bringing patients and providers together for collaborative discussions on information needs to inform the content for inclusion in resources.

The evaluation showed the preferred format for a resource among participants was online and/or paper and both formats have been shown to be effective for patient education [28]. However, through this evaluation there were mixed opinions on how oncofertility resources should be completed and distributed in clinic and who should deliver resources. Some provider participants cited role confusion as a barrier to the provision of fertility information. The 2018 ASCO fertility guidelines target a range of providers, highlighting the need for interdisciplinary involvement in oncofertility counseling and shared decision-making [5]. However, no single provider type is identified as ultimately responsible for discussing FP with patients, which poses a potential challenge in the information delivery process that can result in patients receiving limited or no information and/or conflicting responses between different providers [29]. Effective strategies for the delivery of oncofertility resources with consideration to current care pathways and roles in clinical settings can help ensure patients are receiving resources before making fertility decisions.

The creation and use of oncofertility resources is recommended in the literature to ensure appropriate information delivery to patients, referrals to reproductive specialists, and to improve information retention among patients [7, 8, 10–12, 30–34]. Based on the evaluation results, training for health care providers on oncofertility and shared decision-making may result in a better understanding of patient information needs and value of resources. In addition, we feel the evaluation results should be considered when creating/modifying oncofertility resources and highlight the need for a new Canadian specific resource that adopts the aspects perceived as valuable in existing resources.

However, there are limitations to this evaluation. While provider participants worked in academic and community hospitals across Canada, most patient participants were treated in Ontario potentially limiting the transferability of results to other locations. Participants also included patients who already made their fertility decision, which may have limited their ability to recall what information would have benefited them at the time of decision-making or increased their estimation on the amount of information they would have wanted at the time of their diagnosis based on their current knowledge. However, similar to other studies [35, 36] we did not approach newly diagnosed patients as the review of oncofertility resources from other countries may have caused them to feel conflicted on the relevant information/ fertility options for them in Canada. Additionally, all patient participants had completed at minimum post-secondary education; information needs among low literacy patients may vary from those identified in this evaluation. Planned future work to inform oncofertility resources should aim to recruit individuals with lower literacy [23]. Interviews with patient’s partners were not completed for this evaluation. However, patient’s partners and family generally have some level of involvement in the multiple stages of cancer decision-making [37]. Future research would benefit from the partners perspective and identification of information needs when involved in FP decisions and how they compare to the needs of patients as well as, how partners communicate to mutually come to a fertility decision that is right for their family. Finally, the transferability of results may be limited due to the low overall sample size of participants and the unique nature of information needs for each patient based on their situation and experiences [13, 38]. Despite these limitations, each oncofertility resource was evaluated by at least three participants to help determine the key information that should be considered when creating a resource. Additionally, our research provides important insight into the information needs among young breast cancer patients when making fertility decisions.

## Conclusion

Through the evaluation of existing oncofertility resources by patient and provider participants, the information perceived as important for informed fertility decision-making was identified and summarized. In general, participants perceived the existing oncofertility resources for cancer patients of reproductive age to be useful for informed fertility decision-making. However, there was also information and sections within existing oncofertility resources that participants felt was not necessary for decision-making. While many resources undergo testing throughout development [15, 16], this is the first study that allows patients and providers to view multiple resources allowing for comparisons and a better understanding of how oncofertility content can be delivered in decision aids and patient educational materials. The information reported in this evaluation can be used to inform future development and use of resources for patients facing fertility decisions. Additionally, the evaluation highlighted the differing perceived information needs among patients and providers suggesting that training for health care providers and better communication is required in clinical settings with the use of oncofertility resources to understand specific needs among patients.

## Additional file

Additional file 1: Interview Guide S1. Example interview guide for patient participants (Australian Decision Aid). (DOCX 28 kb)

## Abbreviations

ASCO: American Society of Clinical Oncology
FP: Fertility Preservation
REB: Research Ethics Board
