# Supplementary material for: An evaluation of oncofertility decision support resources among breast cancer patients and health care providers
Source: BMC Health Serv Res. 2019 Feb 6;19:101. doi: 10.1186/s12913-019-3901-z (PMC6366104; doi:10.1186/s12913-019-3901-z)
Supplement: Supplementary file 1 — Interview Guide S1. Example interview guide for patient participants (Australian Decision Aid). (DOCX 28 kb) [file 12913_2019_3901_MOESM1_ESM.docx]

**Additional File 1.** Example interview guide for patient participants (Australian Decision Aid)

**Background**

1. Did you receive surgery, chemotherapy, radiation or hormone therapy following your cancer diagnosis?
2. Did you seek fertility preservation treatment prior to your cancer diagnosis?
3. What was your relationship status when you were diagnosed?
4. Did you have any children when you were first diagnosed?
5. What is your current age?
6. What was your age at diagnosis?
7. Have you finished active treatment in the last 5 years?
8. How would you describe your race/ethnicity?
9. What is the highest grade or year of school you have completed?

**Decision Support Resources**

1. Did you use any decision resources on fertility preservation before you began treatment?
   - 1. If **no** – were you given any information on fertility preservation before you began treatment?
     2. If **yes** – what specific aspects of the resource or information did you find useful?

**Review of Sections in Decision Aid – Australian Decision Aid**

|  | **Not at all useful** | **Not very useful** | **Useful** | **Very useful** | **Not sure** |
| --- | --- | --- | --- | --- | --- |
| 1. How useful do you think the following sections would have been in making a fertility decision? | | | | | |
| *Summary of fertility options* | □ | □ | □ | □ | □ |
| *Some background information* | □ | □ | □ | □ | □ |
| *Fertility-related information* | □ | □ | □ | □ | □ |
| *Clarify the decision* | □ | □ | □ | □ | □ |
| *Compare the options* | □ | □ | □ | □ | □ |
| *Compare how I feel about different options* *(values clarification)* | □ | □ | □ | □ | □ |
| *Determine your decision* | □ | □ | □ | □ | □ |
| *Plan the next steps* | □ | □ | □ | □ | □ |
| *Jenny’s story example* | □ | □ | □ | □ | □ |
| *Some words used in this booklet* | □ | □ | □ | □ | □ |
| *Where to go from here* | □ | □ | □ | □ | □ |
| *Questions to ask your doctors* | □ | □ | □ | □ | □ |
| 1. Overall, how useful do you think this decision resource would have been in helping you decide about fertility options? | □ | □ | □ | □ | □ |

| Decision Support Resource Content and Format | | | | | |
| --- | --- | --- | --- | --- | --- |
|  | **Too short, would prefer it to be much longer** | **Short, would prefer it to be a bit longer** | **Just right** | **Long, would prefer it to be a little shorter** | **Too long, would prefer it to be much shorter** |
| 1. What do you think about the length of the decision resource?   *If* ***(too) long*** *- what sections they believed were too long and why they feel they are not important*  *If* ***(too) short*** *- what sections did they feel were missing from the aid and what other information would be required to make an informed decision* | □ | □ | □ | □ | □ |

|  | **Too few, would prefer a lot more** | **Few, would prefer a few more** | **Just right** | **A lot, would prefer a few less** | **Too many, would prefer a lot less** |
| --- | --- | --- | --- | --- | --- |
| 1. What do you think about the use of graphics in this decision resource?   *What are your thoughts about the risk graphic and photos of people throughout this resource?*  *Are there any sections where figures or illustrations would be more useful to convey the information?* | □ | □ | □ | □ | □ |

|  | **Strongly disagree** | **Disagree** | **Neither agree nor disagree** | **Agree** | **Strongly agree** |
| --- | --- | --- | --- | --- | --- |
| Please indicate how strongly you agree with the following statements | | | | | |
| 1. The information in the decision resource was easy to read | □ | □ | □ | □ | □ |
| 1. The information flows in a logical order | □ | □ | □ | □ | □ |
| 1. I am able to understand the information presented about fertility options | □ | □ | □ | □ | □ |
| 1. The options for fertility preservation were presented equally with no bias   *If* ***(strongly) disagree*** *- what sections did you think were bias? Why?* | □ | □ | □ | □ | □ |
| 1. There is enough information provided to decide which fertility preservation option is right for me | □ | □ | □ | □ | □ |

Utilization of Decision Support Resources

|  | **Strongly disagree** | **Disagree** | **Neither agree nor disagree** | **Agree** | **Strongly agree** |
| --- | --- | --- | --- | --- | --- |
| 1. The decision resource was easy to use | □ | □ | □ | □ | □ |
| 1. Training on how to use the decision resource is needed for patients | □ | □ | □ | □ | □ |
| 1. Training on how to use the decision resource is needed for health care providers | □ | □ | □ | □ | □ |
| 1. I think using this decision resource would do more good for women than harm   *If* ***(strongly) disagree*** *- why do you think it would be harmful for patients to receive this resource?* | □ | □ | □ | □ | □ |
| 1. This decision resource would have been helpful to me in making a decision about fertility preservation had I been able to use it | □ | □ | □ | □ | □ |

1. Would you recommend this decision aid to a friend a similar situation?
2. Why do you think this method of decision making would have worked/wouldn’t have worked for you?

Factors Influencing Fertility Preservation Decisions

|  | **Not at all important** | **Not very important** | **Important** | **Very Important** | **Not sure** |
| --- | --- | --- | --- | --- | --- |
| How important were the following factors when you were considering fertility preservation? | | | | | |
| 1. The stage/severity of diagnosis | □ | □ | □ | □ | □ |
| 1. How much fertility preservation procedures cost | □ | □ | □ | □ | □ |
| 1. That future children are biologically related | □ | □ | □ | □ | □ |
| 1. How much time is required for fertility preservation procedures | □ | □ | □ | □ | □ |
| 1. Are there any other factors that you think are important when considering fertility preservation? | | | | | |
| 1. Which of the above factors would you rank as the top 2 most important factors when you were making fertility preservation decisions? | | | | | |

Delivery and Completion of Decision Support Resources

1. At what time point would you have liked to receive a decision support resource regarding fertility?
2. As soon as you learned about your diagnosis
3. When discussing your treatment plan and before treatment *(what treatment plan?)*
4. During treatment
5. After treatment has been completed
6. Other – *if selected, ask when they would have liked to receive it*
7. How would you have liked to receive a decision support resource regarding fertility?
8. In the mail
9. At an appointment with your surgeon
10. At an appointment with your oncologist
11. At an appointment with a fertility specialist
12. Other – e.g., social workers or nurses
13. How would you have liked to read/complete a decision support resource regarding fertility?
14. With your surgeon
15. With your oncologist
16. With a fertility specialist
17. By yourself
18. Other – e.g., social workers or nurses

*Why would you have preferred to complete a decision support resource this way?*

1. What format do you believe would be the most useful for a decision support resource?
   1. Paper booklet
   2. Online booklet
   3. Interactive online resource
   4. Audio-guide booklet/ video
   5. Other format – *if selected ask what format they believe the decision support resource should be in*

*Why do you think this would be the most useful format for a decision support resource?*
